# Systemic sclerosis: Characterizing candidate retinal and choroidal imaging features using optical coherence tomography

**DOI:** 10.1371/journal.pone.0346705

**Published:** 2026-04-21

**Authors:** Lilla Smeller, Dominika Ördögh, Szilárd Burcsár, Attila Balog, László Szalay, Edit Tóth-Molnár, Nicolette Sohár

**Affiliations:** 1 Department of Ophthalmology, Albert Szent-Györgyi Faculty of Medicine and Pharmacy, University of Szeged, Szeged, Hungary; 2 Department of Rheumatology and Immunology, Albert Szent-Györgyi Faculty of Medicine and Pharmacy, University of Szeged, Szeged, Hungary; University of Warmia, POLAND

## Abstract

**Objectives:**

Systemic sclerosis is a complex autoimmune disease characterized by vasculopathy and fibrosis, capable of affecting virtually any organ system, including the eyes. Optical coherence tomography is a non – invasive imaging technique that enables high-resolution cross-sectional visualization of ocular structures. This study aimed to characterize candidate retinal imaging features in systemic sclerosis patients using Optical Coherence Tomography.

**Methods:**

A total of 41 systemic sclerosis patients (10 males, 31 females; mean age: 58.7 ± 11.7 years) and 38 age-matched healthy controls (9 males, 29 females; 61.6 ± 12.8 years) were included. After all participants underwent a comprehensive ophthalmologic assessment, including best-corrected visual acuity, intraocular pressure measurement, slit-lamp biomicroscopy, fundus examination, and optical coherence tomography was applied.

**Results:**

The mean best-corrected visual acuity among systemic sclerosis patients was 0.95. The central retinal thickness was slightly reduced in systemic sclerosis patients (241.7 µm) compared to controls (254.9 µm), though the difference was not statistically significant. Subfoveal choroidal thickness showed no significant difference (289.9 µm vs. 285.4 µm in controls). Notably, optical coherence tomography revealed previously unreported fundus abnormalities in systemic sclerosis patients: drusen-like deposits in 19 patients (46.3%), wide-based foveal pit in 26 patients (63%), and epiretinal membrane in 9 patients (21%), all of which occurred significantly more often than in the control group (p < 0.01).

**Conclusion:**

Systemic sclerosis is associated with a high prevalence of underrecognized retinal findings, like foveal contour abnormalities and drusen-like deposits. While these candidate imaging features require longitudinal validation, they likely reflect the systemic microvascular and fibrotic burden of the disease.

## Introduction

Systemic sclerosis (SSc) is a chronic immune-mediated connective tissue disease associated with substantial morbidity and reduced quality of life. It primarily affects women and has an estimated annual incidence of 0.6–5.6 per 100,000 adults, with pediatric-onset cases representing fewer than 5% of all patients [1,2]. Prognosis is influenced by several factors, including male sex, older age at onset, diffuse cutaneous subtype, interstitial lung disease (ILD), pulmonary arterial hypertension (PAH), renal and cardiac involvement, digital ulcers, and joint disease [1,3,4].

Clinically, SSc is categorized into limited and diffuse forms based on the extent of skin and internal organ involvement [2,3]. The 2013 classification criteria developed by the American College of Rheumatology (ACR) and the European League Against Rheumatism (EULAR) incorporate both vascular and fibrotic features and emphasize early manifestations such as puffy fingers [5].

The pathogenesis of SSc is multifactorial, involving early endothelial dysfunction, dysregulation of both the innate and adaptive immune systems, and excessive fibroblast activity resulting in progressive fibrosis. The resulting tissue hypoxia and extracellular matrix accumulation contribute to multi-organ damage [2]. Fibrotic skin thickening is a hallmark of SSc and related conditions. Nailfold capillaroscopy is frequently employed to assess peripheral vasculopathy, and progression of skin symptoms often parallels systemic disease advancement [2].

Ocular manifestations of SSc are diverse and may include eyelid abnormalities, dry eye disease, and retinal microvascular changes [6]. Systemic corticosteroids are used during the early stages of SSc. First-line antifibrotic therapy includes mycophenolate mofetil, while emerging treatments involve B-cell depletion (rituximab), IL-6 inhibition (tocilizumab), and tyrosine kinase inhibition (nintedanib) [7].

Optical coherence tomography (OCT) is a non- invasive, high-resolution imaging technique widely used in ophthalmology for quantitative and qualitative evaluation of the retina, choroid, and optic nerve head [8–10].

Although OCT has limitations, such as reduced image quality in cases of low ocular media transparency or poor patient cooperation, it remains a powerful tool in the diagnosis and monitoring of retinal diseases [11,12]. Previous OCT studies in SSc have assessed parameters including choroidal thickness, central macular thickness (CMT), and retinal nerve fiber layer (RNFL) thickness [13–19].

In recent years, efforts to identify new potential OCT markers have intensified, using advanced imaging and molecular techniques. These potential OCT markers might be instrumental in disease monitoring, therapeutic decision-making, and prognostication [20,21].

The aim of this study was to perform OCT examinations in patients with Ssc and evaluate the presence of retinal and choroidal changes that may serve as potential OCT markers. Our findings could provide insight into disease activity, help guide treatment strategies, and ultimately improve visual outcomes in SSc patients.

## Materials and methods

### Study design and participants

This cross-sectional study included patients diagnosed with SSc at the University of Szeged, Hungary.

The inclusion criteria were: (i) a diagnosis of SSc according to the 2013 ACR/EULAR classification criteria, (ii) stable disease for at least 3 months prior to inclusion.

To isolate SSc-specific manifestations and avoid confounding factors, the following exclusion criteria were applied:

1. Inflammatory interference: Any history of previous uveitis or intraocular inflammation that might independently interfere with epiretinal membrane (ERM) formation or retinal thickness.
2. Mechanical/Surgical interference: Previous intraocular surgery (e.g., vitrectomy) orsignificant ocular trauma.
3. Co – morbidities: Diabetes mellitus and uncontrolled systemic hypertension.

A total of 41 SSc patients (10 males, 31 females; mean age: 58.7 ± 11.7 (min: 28, max:78)) were enrolled from 1 March, 2020–31 December, 2023. Thirty-eight age- and sex-matched healthy controls (9 males, 29 females; mean age: 61.6 ± 12.8 (min.: 22, max.: 78)) with no history of ocular disease were also enrolled for comparison.

### Ophthalmological examination

Each participant underwent the following basic ophthalmological examinations performed by an experienced examiner: Best- Corrected Visual Acuity, (BCVA), Intraocular Pressure (IOP), Slit-Lamp Biomicroscopy, Fundus Examination

### Optical coherence tomography (OCT)

OCT imaging was performed using a **Spectralis HRA + OCT** (Heidelberg Engineering, Heidelberg, Germany), which provides high-resolution imaging of retinal and choroidal structures. The following OCT parameters were assessed:

1. **Central Retinal Thickness (CRT):**

CRT was measured from the internal limiting membrane to the retinal pigment epithelium (RPE) at the foveal center using the automated software of the OCT system. A thickness of less than 250 µm was considered as reduced.

1. **Choroidal Thickness (CT):**

Choroidal thickness was measured at the subfoveal region using the EDI mode of the OCT. Measurements were performed manually by the same investigator to ensure consistency.

1. **Subfoveal Choroidal Thickness (SFCT):**

The subfoveal choroidal thickness was determined as the distance from the RPE to the scleral boundary in the central fovea.

1. **Presence of Retinal Abnormalities:**

The OCT scans were analyzed for the presence of macular edema, drusen-like deposits DLD, epiretinal membrane (ERM), and other retinal abnormalities. A trained ophthalmologist reviewed the images to confirm the presence of these abnormalities.

1. **Foveal Contour Abnormality -Wide-based foveal pit (WBFP):**

WBFP is defined as a qualitative loss of the normal acute foveal slope resulting in a broadened and shallower foveal center. To distinguish this from normal variation, a WBFP was characterized by a foveal floor diameter exceeding 400 µm (measured between the foveal crests at the level of the Internal Limiting Membrane) or a visible flattening of the foveal pit slope. To assess foveal contour, OCT B-scans passing through the center of the fovea were analyzed [22].

**Qualitative Assessment:** Two independent, blinded ophthalmologists (LS, NS) evaluated the foveal architecture. A wide-based pit was recorded only if both observers agreed on the departure from normal steep anatomy.

**Quantitative Measurement:** The horizontal distance between the two highest points of the foveal edges (the foveal rim) was measured using the OCT system’s integrated caliper tool.

### Statistical analysis

Data were analyzed by using **SPSS Statistics (version 25, IBM, Armonk, NY, USA)**. Descriptive statistics (mean ± standard deviation) were used to summarize the clinical characteristics and OCT findings. The **Mann-Whitney U test** was used to compare continuous variables between the two groups (SSc patients and healthy controls). Frequencies of the ophthalmological alterations were analysed by Fisher exact test with confidence intervals and odds ratio. Bonferroni adjusted P-value of less than 0.05 was considered statistically significant.

### Ethical considerations

The study adhered to the ethical guidelines for medical research, including the confidentiality of patient data and voluntary participation. Written informed consent was obtained from all participants. Ethical approval was obtained from the local ethics committee at the University of Szeged. This study’s protocol was reviewed and approved by the Regional and Institutional Review Board of Human Investigations at the University of Szeged, approval number is 4693. Date of approval was 21 January, 2020.

## Results

### Demographics and clinical characteristics

A total of 41 SSc patients (10 males, 31 females) with a mean age of 58.7 ± 11.7 years (range: 28–78 years) were included in the study. The mean disease duration of the 41 SSc patients (10 males, 31 females) with a mean age of 58.7 ± 11.7 (range: 28–78 years) years was 11 years (range: 3–30 years). The control group consisted of 38 healthy individuals (9 males, 29 females) with a mean age of 61.6 ± 12.8 years (range: 22–78 years). The age between the two groups showed no statistical difference (P = 0.225).

**Fig 1:**
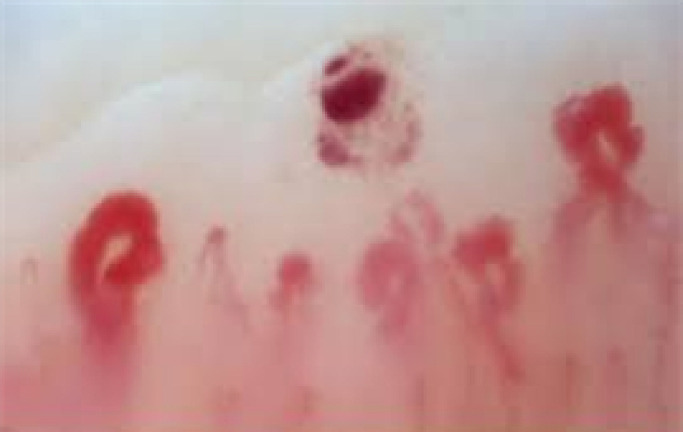
Nailfold capillaroscopy image showing scleroderma pattern: (A) giant capillaries, (B) massively dilated, loop-like vessels, (C) micro-hemorrhages.

Among the SSc patients, 25 (60%) had the limited cutaneous form of SSc, while 16 (39%) had the diffuse form. Laboratory tests showed that 38 patients (92.6%) tested positive for antinuclear antibodies (ANA), 18 patients (43.9%) had anti-SCL-70 positivity, and 15 patients (36.5%) were positive for anti – centromere antibodies. All patients had Raynaud’s phenomenon, 29 patients (70.7%) had skin involvement, and 11 patients (26.8%) had digital ulcers. Fig 1. presents a capillaroscopy picture of a SSc patient.

Pulmonary fibrosis and pulmonary hypertension were present in 22 (53.6%) and 7 (17.07%) patients, respectively. Gastrointestinal involvement and hypertension were found in 34 (82.9%) and 6 patients (14.6%), respectively. The demographic and clinical data of the 41 patients are shown in Table 1.

**Table 1:** Demographics and Systemic Charasteristics of SSc Patients.

Characteristic	SSc Patients (n = 41)	Healthy Controls (n = 38)	p-value
**Demographics**			
Age (years), mean	58.7 ± 11.7	61.6 ± 12.8	0.225
Gender (Male/Female), n (%)	10/31 (24%/75.6%)	9/29 (22%/70%)	>0.99
Disease duration (years), mean (range)	11 (3-30)	N/A	
**SSc Subtype**			
Limited cutaneous (lcSSc), n (%)	25(60%)	N/A	
Diffuse cutaneous (dcSSc), n (%)	16 (39%)	N/A	
**Autoantibody Profile, n (%)**			
Antinuclear Antibodies (ANA)	38/41 (92.7%)	N/A	
Anti-Scl-70 (Topoisomerase I)	18/41 (43.9%)	N/A	
Anti-centromere (ACA)	15/41 (36.6%)	N/A	
**Organ Involvement, n (%)**			
Raynaud’s phenomenon	41/41 (100%)	0/38 (0%)	
Skin involvement (mRSS > 0)	29/41 (70.7%)	0/38 (0%)	
Digital ulcers (history/present)	11/41 (26.8%)	0/38 (0%)	
Interstitial Lung Disease (ILD)	22/41 (53.6%)	0/38 (0%)	
Pulmonary Arterial Hypertension	7/41 (17.0%)	0/38 (0%)	
Gastrointestinal involvement	34/41 (82.9%)	0/38 (0%)	
Systemic Hypertension	6/41 (14.6%)	3/38 (7.9%)	

### Best corrected visual acuity (BCVA)

The mean best-corrected visual acuity (BCVA) in the SSc patient group was 0.95 ± 0.11 (range: 0.03–1.0), while in the healthy control group, the mean BCVA was 0.95 ± 0.11 (range: 0.65–1.0). No significant difference was found in BCVA between the two groups.

### OCT findings: Retinal and choroidal thickness

- **Central Retinal Thickness (CRT)**

The mean central retinal thickness in SSc patients was 241.7 ± 22.9 µm (range: 200–314 µm), which was slightly lower than the mean CRT of 254.9 ± 39.2 µm (range: 190–410 µm) in healthy controls, but the difference was not statistically significant (P = 0.119).

- **Subfoveal Choroidal Thickness (SFCT)**

The mean SFCT in SSc patients was 289.9 ± 65.9 µm (range: 140–475 µm), compared to 285.4 ± 41.3 µm (range: 220–402 µm) in healthy controls. The difference between the groups was not statistically significant (P = 0.465).

### Fundus abnormalities detected by OCT

Several previously unreported retinal pathologies were also observed in SSc patients:

- **Drusen-like deposits** (DLD)

**Fig 2:**
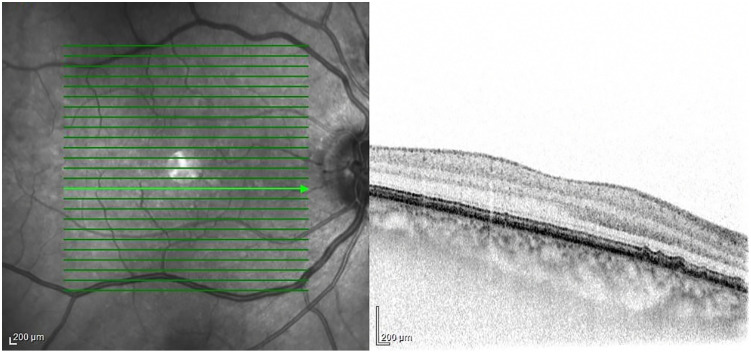
OCT image from the right eye of a 68-year-old male SSc patient. DLD are located between the basal lamina of the retinal pigment epithelium (RPE) and the inner collagenous layer of Bruch’s membrane (BM).

Drusen-like deposits (DLD) were detected in 19 SSc patients (46.3%) (Fig 2). This was significantly higher than the prevalence of drusen in the healthy control group (13%) (P = 0.0015).

- **Foveal Contour Abnormality - Wide-based foveal pit (WBFP)**

**Fig 3:**
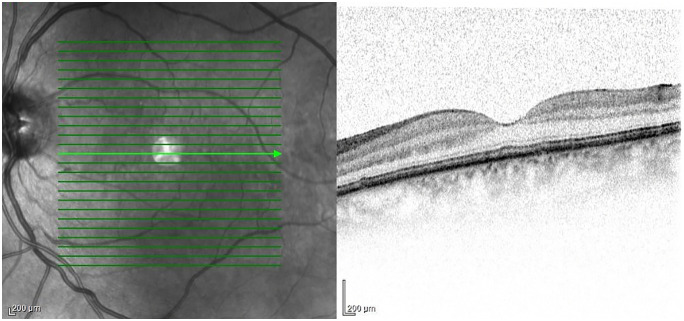
OCT image from the left eye of a 45-year-old female SSc patient. WBFP can be observed.

WBFP was observed in 26 SSc patients (63%) (Fig 2), which was also significantly more frequent than in the control group (0%) (P < 0.0001) Fig 3 and Table 2.

**Table 2:** Morphological Analysis of Foveal Architecture.

Feature	SSc Patients (n = 41)	Controls (n = 38)	P-value
Normal Foveal Pit	15 (36.6%)	38 (100%)	<0.0001
Wide-based Foveal Pit	26 (63.4%)	0 (0%)	<0.0001
Mean Foveal Width (µm)	482.5 ± 42.1	312.4 ± 28.6	<0.001

- **Epiretinal Membrane (ERM)**

**Fig 4:**
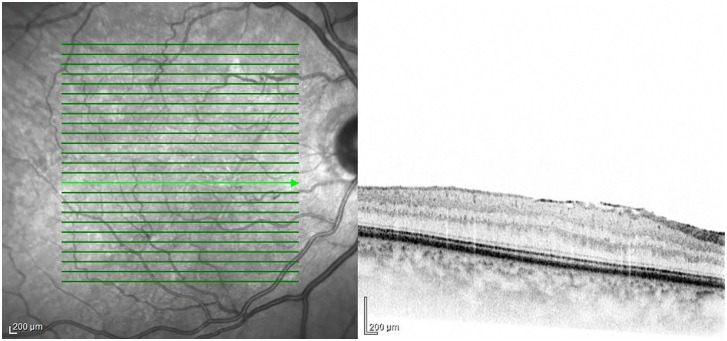
OCT image of the ERM of a 48-year-old female SSc patient. A reflective layer is visible on the top of the internal limiting membrane (ILM) of the left eye in a 34-year-old patient. The ERM is attached to the retinal surface.

ERM was identified more frequently in SSc patients (9 cases, 21%) (Fig 4), than in the control group (1 case, 2.4%), the difference was not significant (P = 0.225).

- **Retinal Vasculopathy**

**Fig 5:**
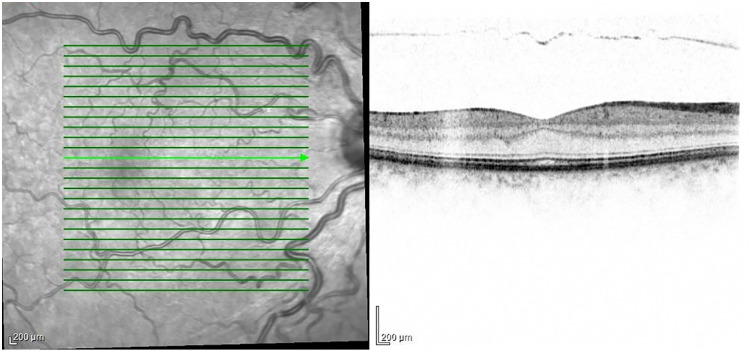
OCT image from the right eye of a 57-year-old male SSc patient. (A) Increased vascular tortuosity, (B) general arteriolar narrowing, (C) arteriovenous notch are visible.

Retinal vasculopathy, including vascular tortuosity, arteriolar narrowing, and arteriovenous notches, was observed in 26 SSc patients (63.4%) (Fig 4). These findings were consistent with known signs of vasculopathy in SSc, and they appeared in patients both with and without hypertension. This finding was significantly more common than in the control group (8%) (P <0.0001) Fig 5.

### Other retinal findings

Additional retinal pathologies were noted in a small number of SSc patients: choroidal nevus: 4 patients (9.7%), myopic fundus: 2 patients (4.8%), fundus dystrophy: 1 patient (2.4%), age-related macular degeneration (AMD): 3 patients (7.9%)

The ophthalmological parameters of the 41 SSc patients are presented in Table 3. All original data are presented in supporting files (S1 and S2 Tables).

**Table 3:** Comparison of Ophthalmological Parameters.

Parameter	SSc (n = 41)	Control (n = 38)	Bonferroni adjusted P	Odds Ratio	95% CI
BCVA (mean)	0.95	0.95	1		
IOP (mmHg)	14,80	16,30	0.75		
CRT (µm)	241,7	254,9	1		
SFCT (µm)	289,9	285,4	1		
DLD/drusen n (%)	19 (46%)	5 (12%)	**0,0225**	5,279	1.781, 15.649
ERM n (%)	9 (22%)	1 (2%)	0,225	7,308	1.226, 43.549
Wide-based Foveal Pit n (%)	26 (63%)	0	**<0.0001**	131,645	7.544, 2297.196
Vasculopathy n (%)	26 (63%)	3 (7%)	**<0.0001**	17,341	4.897, 61.402

CI: Confidence intervals.

### Statistical comparison

The occurrence of DLD and WBFP was significantly higher in SSc patients compared to the healthy control group (P < 0.05 for all comparisons). The incidence of retinal vasculopathy was also significantly elevated in SSc patients (63.4%) compared to controls (P < 0.0001). ERM was found more frequently in SSC cases than in the control group, but the difference was not significant (P = 0.225).

## Discussion

The pathogenesis of SSc is complex, involving generalized vasculopathy, an abnormal immune response, and fibrosis due to excessive fibroblast activity and extracellular matrix protein deposition [23]. These pathological processes can lead to tissue hypoxia, affecting both superficial and deeper ocular structures. Although SSc diagnoses and outcomes are primarily clinically defined, there remains significant interest in identifying potential OCT markers that could assist in monitoring disease activity, guiding treatment, and predicting clinical outcomes [24].

In recent years, early diagnosis of scleroderma has been the subject of intensive research, including new non-invasive testing methods. Close monitoring of potential multi-organ involvement is also essential. Subclinical abnormalities can fundamentally determine the outcome of the disease, including cardiac and pulmonary involvement and small and large vessel vasculopathies. Continuous, mostly low-grade inflammation and progressive fibrosis are key factors of pathological changes in scleroderma, including eye symptoms. Identifying these subtle or non-specific signs of symptoms is crucial for potential early intervention and preventing organ damage [19–21,23].

Ocular involvement in SSc has been described in various studies, often focusing on eyelid abnormalities, ocular surface disease, and retinal microvascular changes [6]. The ophthalmic manifestations of SSc are not always clinically evident and, as a result, there is increasing interest in non-invasive imaging techniques such as OCT to explore potential early signs of ocular involvement [17,25,26].

By strictly excluding cases of previous uveitis, we have demonstrated that the increased prevalence of ERM and foveal contour changes is likely a primary manifestation of SSc-related fibrosis rather than a secondary post-inflammatory effect. Using the Govetto framework, we observed a **wide-based foveal pit** in 63.4% of SSc patients. We hypothesize that systemic pro-fibrotic cytokines stimulate glial proliferation at the vitreoretinal interface. This glial activation exerts centripetal mechanical forces, broadening the foveal contour. This morphological change may represent an early stage of vitreoretinal remodeling before the clinical appearance of a distinct ERM [22].

In this study, we observed several new and previously unreported ocular findings, including DLD and WBPF in SSc patients. These findings were significantly more common in the SSc group compared to healthy controls, underscoring the importance of OCT in the early detection of ocular complications that may affect visual acuity and overall prognosis.

Chronic low-grade inflammation promotes microvascular obliteration. This ischemic environment impairs the metabolic clearance of the Retinal Pigment Epithelium (RPE), leading to the accumulation of extracellular deposits observed as DLD in 46.3% of our cohort. DLD, which are extracellular deposits found between the retinal pigment epithelium (RPE) and Bruch’s membrane, were detected in 46.3% of SSc patients. Drusen are typically associated with AMD [27,28]. DLD are morphologically similar to drusen but etiologically distinct.

The WBFP (63.4%) may serve as an early sentinel sign of systemic fibrotic progression. Unlike thickness parameters (SFCT, CRT), which showed high variability, these qualitative morphological changes provide a more specific window into the disease’s impact on highly metabolic ocular tissues.

In order to see the correlation between SSc dubgroups, limited vs diffuse; between ophthalmological findings and duration, between pulmonary findings and OCT findings, we could state that:

This study found no significant differences in retinal thickness or choroidal thickness when comparing Limited Cutaneous SSc (lcSSc) to Diffuse Cutaneous SSc (dcSSc). SSc Type Influence: Diffuse SSc + ERM: 4 out of 5 (80%) have ILP. Limited SSc + ERM: Only 1 out of 4 (25%) have ILP. Both subtypes generally showed significant thinning of the retina and choroid compared to healthy controls, but the subtype itself did not predict the severity of the ocular thinning. This suggests that the microvascular damage in the eye occurs early and is a common feature of the systemic disease regardless of the extent of skin involvement.

There is no strong or statistically significant correlation between ERM and disease duration. Presence of ERM appears to be independent of how long a patient has had SSc.

Correlation: ILP prevalence: within the ERM group, 55.6% (5 out of 9) have ILP. This is nearly identical to the 53.1% prevalence found in patients without ERM.

Lung involvement (ILP) is a very frequent finding in this group (affecting more than half the patients). Because it is so widespread across all durations and types, isolated ocular findings like ERM aren’t specific enough to distinguish who will have it.

DLD Comparison: DLD has a much stronger association with PAH than ERM has with anything. Patients with DLD in our dataset are nearly 3 times more likely to have PAH than those without it, though the overall sample size remains small. If we are looking for a potential ocular marker for systemic severity in this group, DLD has a much stronger (though still modest) association with PAH than ERM does. ILP is so prevalent in SSc patients that neither ocular marker serves as a meaningful differentiator for it. These structrural abnormalities were observed across the full spectrum odf disease chronicity, suggesting that ocular remodeling may occur early in the disease course.

Since Fundus Autofluorescence (FAF) or AREDS grading were not used, to differentiate SSc-associated deposits from typical age-related drusen, we applied the following criteria:

*Distribution*: Deposits were assessed for their ‘scattered’ or ‘extramacular’ distribution, which is less common in early AMD.

*Morphology:* We looked for cuticular-like patterns (small, “saw-tooth” elevations) or Basal Laminar Deposits (BLamD), which appear as a diffuse thickening of the RPE-Bruch’s membrane complex.

*Control Comparison:* The significantly lower prevalence in the age-matched control group (13%) suggests that the 46.3% observed in the SSc group is not merely a function of chronological aging.

The high prevalence of sub-RPE deposits in our relatively young SSc may reflect premature RPE senescence secondary to chronic choroidal ischemia. Unlike the lipid-rich drusen of AMD, these deposits may represent Basal Laminar Deposits, a recognized consequence of impaired oxygenation from the underlying choriocapillaris, which is a hallmark of systemic sclerosis-related microangiopathy [27–29].

The formation of DLD on the retina of SSc patients is primarily associated with microvascular damage and ischemia caused by the systemic vascular pathology characteristic of SSc. This leads to obliteration or narrowing of small retinal blood vessels, resulting in compromised blood flow and hypoxia. This ischemic environment can promote the accumulation of extracellular deposits, forming DLD. Endothelial cell injury and dysfunction in SSc contribute to impaired clearance and increased deposition of metabolic waste, fostering DLD development. Chronic immune activation and inflammation related to SSc can contribute to remodeling of tissue and deposit formation under the retina [27,28].

In order to differentiate DLD in SSc from drusen in AMD, we have to know, that drusens in AMD are located in the macula, and may progress to geographic atrophy or choroidal neovascularization. SSc associated with DLD may be numerous, bilateral, and scattered; found outside the macula; stable over time; and not linked to vision loss. Fluorescein angiography may show leakage or choroidal neovascularisation in cases of AMD and vascular irregularities with systemic microangiopathy in SSc patients [29].

In our cohort, only three patients exhibited signs of AMD, suggesting that DLD formation in SSc may not be directly linked to degenerative macular diseases but rather to the underlying fibrotic processes in the eye of SSc patients.

ERM formation in SSc is driven by systemic vascular injury, hypoxia-induced cellular proliferation, and fibrosis, leading to the contraction and development of a membrane on the retinal surface [30,31].

The presence of ERM is particularly concerning as it is associated with visual impairment, and previous studies have shown that secondary ERMs can lead to worse visual outcomes compared to idiopathic ERMs [30,31]. The higher prevalence of ERM in SSc patients could represent a previously underappreciated complication of the disease, which may warrant closer monitoring. There was significant correlation between the presence of DLD and the duration of the disease (p < 0.05) but not between the presence of ERM and the duration of the disease (p > 0.05).

### Limitations of the study and future directions

While this study provides valuable insights into the ocular manifestations of SSc, it is not without limitations. The sample size is relatively small, and further studies with larger cohorts are needed to confirm the findings and better understand the underlying mechanisms.

This study is hypothesis-generating and limited by its cross-sectional design. A limitation of this study is the lack of adjustment for axial length, which can influence the lateral magnification of OCT scans. While this does not invalidate the qualitative findings (DLDs, foveal contour), it may introduce variability in the quantitative thickness measurements. The cross-sectional design prevents us from establishing a causal or predictive relationship between the identified retinal features and systemic disease progression. Second, we did not perform correlations with specific clinical severity scores, such as the Modified Rodnan Skin Score (mRSS) which limits the ability to classify these findings as validated clinical biomarkers.

The impact of treatment exposure (e.g., immunosuppressants or antifibrotics) on these retinal structures remains unknown.

Longitudinal studies are now required to determine if these retinal markers can predict systemic organ involvement, such as pulmonary fibrosis.

## Conclusions

In this study, we identified new and previously unreported potential OCT markers in patients with SSc, including DLD and WBFP. These abnormalities were found to occur significantly more frequently in SSc patients compared to age-matched healthy controls, suggesting that they may serve as important indicators of ocular involvement in this autoimmune disorder.

Our findings suggest that OCT may serve as a sentinel tissue for systemic fibrotic and vascular processes in SSc. While the identified features—drusen-like deposits and wide-based foveal pits—are not yet validated diagnostic biomarkers, they represent candidate imaging markers for subclinical ocular involvement. This study is hypothesis-generating, establishing a foundation for longitudinal trials to determine if these retinal changes parallel systemic organ progression.

Future research should focus on longitudinal monitoring to calculate the Positive Predictive Value (PPV) of a WBFP for systemic fibrotic flares.

In conclusion, the findings of this study underscore the utility of OCT in identifying new retinal morphological associations (DLD and WBFP) in SSc. These candidate imaging features reflect the dual burden of fibrosis and vasculopathy. Routine OCT screening is recommended as a non-invasive method to monitor multi-organ involvement in SSc.

## Supporting information

S1 Table Ophthalmological data of SSc patients. (XLSX)

S2 Table Ophthalmological data of controls. (XLSX)
